# Association of VitD 3 deficiency with thyroid nodules suspected of malignancy in petroleum workers: a retrospective cohort study

**DOI:** 10.7717/peerj.20893

**Published:** 2026-02-27

**Authors:** Deping Wang, Dongdong Luo, Song Leng, Bingrui Gao, Chenxi Zhang, Zhaoying Chen, Bojuan Li, Jialin Hu, Zhongyan Shan, Weiping Teng, Jing Li

**Affiliations:** 1 Department of Endocrinology and Metabolism, Hongqi Hospital Affiliated to Mudanjiang Medical University, Mudanjiang, Heilongjiang, China; 2 Department of Endocrinology and Metabolism, The Institute of Endocrinology, NHC Key Laboratory of Diagnosis and Treatment of Thyroid Disease, The First Hospital of China Medical University, Shenyang, Liaoning, China; 3 Department of Endocrinology and Metabolism, The Second Hospital of Dalian Medical University, Dalian, Liaoning, China; 4 Health Management Center, The Second Hospital of Dalian Medical University, Dalian, China; 5 Liaoning University of Traditional Chinese Medicine, Shenyang, Liaoning, China

**Keywords:** Thyroid nodules, Ultrasound, 25(OH)VD, 25(OH)VD2, 25(OH)VD3, C-TRIADS

## Abstract

**Background:**

Thyroid nodules (TNs) are common thyroid disorders. Vitamin D (VitD) is linked to thyroid disease risk, but prior studies mainly focused on total serum VitD and TN risk, ignoring different impacts of VitD3 and VitD2 metabolites on TN development.

**Methods:**

Between July and December 2021, we conducted a retrospective cohort study in Dalian, China, involving 2,037 euthyroid male petroleum workers (aged 30–60 years) without thyroid autoimmunity (TAI). Serum 25-hydroxy vitamin D [25(OH)VD], 25(OH)VD3 and 25(OH)VD2 levels were assayed by liquid chromatography-tandem mass spectrometry (LC-MS/MS). The participants were classified into different groups according to their ultrasound images of TNs based on Chinese-Thyroid Imaging Reporting and Data System (C-TIRADS). C-TIRADS consist of 6 grades, and the upper three indicate TNs with potential malignancy.

**Results:**

Analysis of the cohort revealed the prevalence of TNs and VitD levels in this population. No C-TIRADS 5 or 6 nodules were identified. Serum 25(OH)VD levels in the C-TIRADS 4 TN group were significantly lower than those in the C-TIRADS 1, 2, and 3 groups. The overall prevalence of TNs was similar among the VitD-deficient, insufficient, and sufficient groups. However, the prevalence of patients with C-TIRADS 4 TNs was markedly higher in the VitD-deficient group (18.5%) than in the insufficient (5.5%, *p* < 0.001) and sufficient groups (4.1%, *p* < 0.001). Identical findings were observed among the groups with low, medium, and high serum 25(OH)VD3 levels, but not among those with different serum 25(OH)VD2 levels. Binary logistic regression analysis revealed that low VitD3 levels [25(OH)VD3 < 19.07 ng/mL] were associated with a significantly increased risk of developing C-TIRADS 4 TNs, with an adjusted odds ratio (OR) of 4.74 (95% confidence interval (CI) [3.13–7.18]; *p* < 0.001), compared to high VitD3 levels, after adjusting for confounding variables such as age, body mass index (BMI), thyroid function, thyroid autoantibodies, and season of blood draw.

**Conclusions:**

VitD3 deficiency, but not VitD2 deficiency, was independently associated with TNs with suspicious malignancy in euthyroid male petroleum workers aged 30–60 years.

## Introduction

Thyroid nodules (TNs) represent a highly prevalent thyroid condition, exhibiting an overall prevalence of approximately 25% in the general population (Mu et al., 2022). The widespread adoption of sensitive imaging modalities, particularly in neck assessments, has facilitated the frequent incidental detection of TNs, with up to 60% of adults harboring at least one such nodule (Durante et al., 2023). Although the risk of malignancy drives clinical assessment, the overall cancer prevalence in unselected TNs remains low, typically ranging between 1% and 5% (Grussendorf, Ruschenburg & Brabant, 2022). Against this backdrop of high prevalence, understanding the pathogenesis of TNs and related contributing factors—especially those implicated in thyroid carcinogenesis—has become a critical focus of contemporary research.

Among the various factors implicated in TNs, vitamin D (VitD) has garnered increasing research interest, alongside established risk factors including iodine deficiency, female sex, obesity, metabolic syndrome, and exposure to head or neck ionizing radiation (Grani et al., 2024; Ringel et al., 2025). Nevertheless, the relationship between VitD status and malignant TNs remains particularly ambiguous. Several studies report that lower circulating 25-hydroxy vitamin D (25(OH)VD) levels correlate with an increased incidence of malignant TNs (Abdellateif et al., 2020; Bains et al., 2021; Li et al., 2025; Shi et al., 2025), while others find no significant association (Jonklaas, Danielsen & Wang, 2013; Unlu et al., 2023; Lanitis et al., 2025). Prognostic relevance is similarly debated: some studies link lower preoperative 25(OH)VD levels to adverse clinicopathologic features in papillary thyroid carcinoma (PTC) (Kim et al., 2014; Sulibhavi et al., 2019), whereas others have failed to establish an association between low VitD levels and PTC invasiveness, such as multicentricity (defined as multiple tumor foci within the thyroid gland), lymphovascular invasion, and metastasis (Demircioglu et al., 2022; Alfaleh et al., 2025). Equally unresolved is the association between VitD and benign TNs: one study reports an inverse correlation between VitD levels and benign TN prevalence in euthyroid individuals (Bolat & Erdoğan, 2022), while another finds no elevated benign TN risk among VitD-deficient centenarians (Fan et al., 2022; Li et al., 2025).

A critical oversight in existing research may explain these inconsistencies: the neglect of differential effects between VitD’s two primary forms, VitD3 (cholecalciferol) and VitD2 (ergocalciferol), which exhibit profound differences in biological activity (Dutta et al., 2025). Though sharing a four-ring sterol core, they differ structurally: VitD2 contains a C24 methyl group and C22-C23 double bond absent in VitD3 (Bikle, 2014), altering molecular conformation and thereby affecting binding to VitD binding protein (DBP), affinity for the VitD receptor (VDR), and metabolic fate (Dutta et al., 2025). VitD3—primarily derived from animal sources or cutaneous synthesis *via* UV irradiation—binds DBP more strongly, enhancing circulatory stability and tissue uptake, whereas plant-sourced VitD2 undergoes less efficient conversion to active metabolites, weaker DBP binding, and faster clearance (Chun et al., 2016). Hepatic CYP2R1, a key VitD 25-hydroxylase, preferentially hydroxylates VitD3 (Chun et al., 2016). The resulting active metabolite, 1,25-dihydroxyvitamin D3 (1,25(OH)_2_VD3), retains its VDR-binding capacity even following subsequent 24-hydroxylation (forming 1,24,25-trihydroxyvitamin D3 (1,24,25(OH)_3_VD3)). This stands in contrast to VitD2, where the 24-hydroxylation of its active metabolite produces 1,24,25-trihydroxyvitamin D2 (1,24,25(OH)_3_VD2), which is fully inactivated (Tripkovic et al., 2012). Importantly, VDR activation is critical for VitD function, and VitD3 metabolites exhibit higher VDR affinity than VitD2 metabolite, enabling more robust activation of downstream signaling pathways (Horst et al., 2000). Collectively, these differences support VitD3 as the more biologically active form (Tripkovic et al., 2012), yet existing studies focus almost exclusively on total serum VitD, ignoring potential divergent effects of VitD3 and VitD2 on TN development. Portions of this text were previously published as part of a preprint (Wang et al., 2023).

To address these gaps, we conducted a retrospective analysis investigating associations between serum levels of total VitD, VitD3, and VitD2 and overall TN prevalence, with particular emphasis on nodules exhibiting malignant ultrasound features.

## Materials & Methods

### Study design

**Figure 1:**
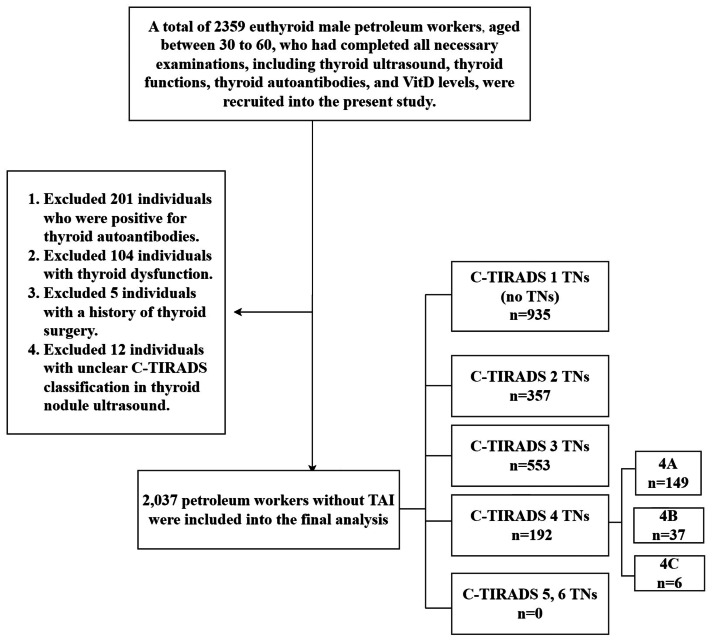
Flow chart of the study screening process. Abbreviations: C-TIRADS, Chinese Thyroid Imaging Reporting and Data System; TAI, thyroid autoimmunity; TNs, thyroid nodules.

A retrospective analysis was carried out utilizing data from the Second Hospital of Dalian Medical University. The focus of this analysis was on the health check-ups of euthyroid male petroleum workers spanning from July 2021 to December 2021. At the beginning, 2,359 workers aged between 30 and 60 who had successfully completed all the required examinations were recruited for this study. These examinations included thyroid ultrasound, thyroid function tests, thyroid autoantibody tests, and measurements of VitD levels. All participants had no history of intestinal diseases that could potentially affect serum VitD levels, such as celiac disease, malabsorption syndromes, or a history of small intestine resection (Ahn et al., 2023). Moreover, none of the participants were taking medications that could influence serum calcium or VitD levels, like VitD analogs, glucocorticoids, or osteoporosis medications. Among them, 201 workers with positive thyroid autoantibodies, 104 with thyroid dysfunction, 5 with a history of thyroidectomy, and 12 with unclear Chinese-Thyroid Imaging Reporting and Data System (C-TIRADS) ultrasound classification of TNs were excluded (Fig. 1). All participants provided written informed consent for study participation. The Ethics Committee of the Second Hospital of Dalian Medical University approved the study protocol (Ethical Application Ref: 2022/090). This retrospective cohort study was conducted and reported in accordance with the Strengthening the Reporting of Observational Studies in Epidemiology (STROBE) guidelines.

Given that low serum VitD is an identified independent risk factor for Hashimoto’s thyroiditis (Giovinazzo et al., 2017; Ke et al., 2017; De Pergola et al., 2018), and thyroid autoantibodies are closely linked to thyroid cancer (Chen et al., 2013; Feldt-Rasmussen, 2020), we specifically selected petroleum workers without thyroid autoimmunity (TAI) as our final study population to minimize confounding. Female participants were excluded due to their significant underrepresentation within the petroleum industry workforce, preventing meaningful gender-stratified analysis and avoiding potential gender-related confounding.

### Research sample

A total of 2,037 petroleum workers without TAI were included in the subsequent analysis. The TNs were classified into six grades according to the C-TIRADS criteria. Participants were grouped according to the highest C-TIRADS classification of TNs shown in their ultrasound results. Among the participants, 935 had no TNs (C-TIRADS 1 nodules), 357 had C-TIRADS 2 nodules, 553 had C-TIRADS 3 nodules, and 192 had C-TIRADS 4 nodules. The 192 participants with C-TIRADS 4 nodules were further divided into 149 with 4A, 37 with 4B, and 6 with 4C. However, no C-TIRADS 5 or 6 nodules were detected in these participants (Fig. 1).

### Laboratory measurements

Serum concentrations of thyroid stimulating hormone (TSH), free triiodothyronine (FT3), free thyroxine (FT4), antithyroperoxidase antibody (TPOAb), and antithyroglobulin antibody (TgAb) were measured *via* chemiluminescent enzyme immunoassays (Siemens CENTAUR XP). Concentrations of 25(OH)VD3 and 25(OH)VD2 were quantified by liquid chromatography-tandem mass spectrometry (LC-MS/MS; AB SCIEX Triple Quad™ 4500MD), in compliance with the China National Accreditation Service for Conformity Assessment (CNAS) requirements for *in vitro* diagnostic medical devices. For 25(OH)VD3, the maximum acceptable dilution factor was 8, with a linear range of 3.13–100.00 ng/mL and a clinical reportable range of 3.13–800.00 ng/mL; precision evaluations at high concentrations yielded coefficients of variation (CV) of within-run = 1.92%, between-run = 2.22%, and within-laboratory = 2.71%, while at low concentrations, the values were within-run = 1.88%, between-run = 1.44%, and within-laboratory = 2.11%. Similarly, for 25(OH)VD2, the maximum dilution factor was 8, with a linear range of 0.78–25.00 ng/mL and a clinical reportable range of 0.78–200.00 ng/mL; precision at high concentrations showed within-run CV = 2.74%, between-run CV = 2.92%, and within-laboratory CV = 3.68%, and at low concentrations, within-run CV = 3.64%, between-run CV = 2.12%, and within-laboratory CV = 3.65%. Total serum 25(OH)VD was calculated as the sum of 25(OH)VD3 and 25(OH)VD2.

Thyroid autoantibody positivity was defined as TPOAb > 60.00 IU/mL and/or TgAb > 60.00 IU/mL, with results otherwise classified as negative. Based on international and domestic guidelines (Holick et al., 2011), total 25(OH)VD levels were categorized as deficient (VD-L, <20.00 ng/mL), insufficient (VD-M, 20.00–29.99 ng/mL), or sufficient (VD-H, ≥ 30.00 ng/mL). As optimal reference ranges for 25(OH)VD3 and 25(OH)VD2 individually are not established, concentrations were stratified using tertiles: 25(OH)VD3 levels were classified as low (VD3-L, <19.07 ng/mL), medium (VD3-M, 19.07–26.69 ng/mL), or high (VD3-H, ≥ 26.70 ng/mL), while 25(OH)VD2 levels were classified as low (VD2-L, <0.49 ng/mL), medium (VD2-M, 0.49–0.99 ng/mL), or high (VD2-H, ≥ 0.99 ng/mL). Tertile ranges were determined based on the distribution of 25(OH)VD3 and 25(OH)VD2 concentrations in the study population (25(OH)VD3: 25th percentile = 19.07 ng/mL, 75th percentile = 26.70 ng/mL; 25(OH)VD2: 25th percentile = 0.49 ng/mL, 75th percentile = 0.99 ng/mL).

### Statistical analysis

The statistical analyses were performed using SPSS Statistics 27.0 (SPSS, Inc.). Continuous variables with normal distributions, such as FT3 and FT4, are presented as mean ± standard deviation (SD). Non-normally distributed variables, including TSH, body mass index (BMI), age, 25(OH)VD, 25(OH)VD3, and 25(OH)VD2, are summarized as median (interquartile range, Q25–Q75). For normally distributed data with homogeneous variances (FT3 and FT4), one-way analysis of variance (ANOVA) was employed. The non-parametric Kruskal–Wallis *H* test was conducted for non-normally distributed data or data with heterogeneous variances (TSH, BMI, age, 25(OH)VD, 25(OH)VD3, and 25(OH)VD2). Categorical variables were compared using the chi-square test, with multiple comparisons adjusted *via* the Bonferroni correction method. Statistical significance was defined as an adjusted *p*-value (Padj) < 0.05, calculated using a Bonferroni correction factor of three applied to the raw *p*-value. Finally, binary logistic regression analysis was performed to estimate the odds ratios (ORs) and 95% confidence intervals (CIs) for the risk of C-TIRADS 4 TNs associated with different levels of VitD.

## Results

### Demographic characteristics and clinical parameters comparison

Analysis of health examination data from 2,037 participants revealed that among petroleum workers without TAI, 54.1% (1,102/2,037) had TNs, while 9.4% (192/2,037) were classified as C-TIRADS 4 TNs by ultrasound. Significant differences existed in age, FT4, FT3, and TSH levels across C-TIRADS categories (*p* < 0.05), though no differences were observed for BMI or season of blood draw (*p* > 0.05; Table 1). The TIRADS 3 TN group exhibited slightly lower TSH levels than the no-TN group. Conversely, other groups showed no significant TSH differences compared to the no-TN group (Table 1). Serum concentrations of 25(OH)VD and 25(OH)VD3 were significantly lower in the C-TIRADS 4 TN group *versus* C-TIRADS 1–3 groups (*p* < 0.001). However, 25(OH)VD2 concentrations did not differ significantly among groups (*p* > 0.05; Table 1). Additionally, the C-TIRADS 4 TN group had a significantly higher prevalence of VitD deficiency [25(OH)VD < 30.00 ng/mL] (89.1%) than the C-TIRADS 1 (74.0%; *p* < 0.001), C-TIRADS 2 (75.1%; *p* < 0.001), and C-TIRADS 3 (71.6%; *p* < 0.001) cohorts (Table 1).

**Table 1:** Comparison of demographic and clinical parameters in 2,037 petroleum workers with TNs by C-TIRADS classification.

	Total	C- TIRADS 1 TNs group	C-TIRADS 2 TNs group	C- TIRADS 3 TNs group	C-TIRADS 4 TNs group	*p*-value
n	2,037	935	357	553	192	
Age (years)	50.00 (45.00–54.00)	48.00 (43.00–52.00)	51.00^a^ (46.00–55.00)	51.00^a^ (47.00–56.00)	51.00^a,b,c^ (48.00–56.00)	<0.001
BMI (kg/m2)	25.73 (23.78–28.03)	25.66 (23.62–27.99)	25.61 (23.74–27.73)	25.72 (23.89–28.04)	26.26 (24.22–28.74)	0.061
FT3 (pmol/L)	5.32 ± 0.43	5.34 ± 0.42	5.28 ± 0.45	5.30 ± 0.43	5.36 ± 0.433	0.019
FT4 (pmol/L)	16.43 ± 1.87	16.32 ± 1.82	16.34 ± 1.91	16.44 ± 1.84	17.07 ± 1.97^a,b,c^	<0.001
TSH (mIU/L)	1.36 (1.02–1.84)	1.41 (1.07–1.91)	1.36 (1.00–1.89)	1.30^a^ (0.91–1.73)	1.30 (1.02–1.82)	<0.001
25(OH)VD (ng/mL)	23.97 (18.05–30.01)	23.68 (18.04–30.19)	24.65 (19.33–29.82)	25.06^a^ (19.94–30.79)	17.59^a,b,c^ (12.64–23.72)	<0.001
25(OH)VD3 (ng/mL)	22.77 (17.14–28.82)	22.67 (17.11–29.04)	23.56 (18.31–28.65)	24.41^a^ (18.65–30.02)	16.55^a,b,c^ (11.74–22.49)	<0.001
25(OH)VD2 (ng/mL)	0.70 (0.40–1.29)	0.71 (0.40–1.38)	0.67 (0.40–1.25)	0.67 (0.40–1.22)	0.76 (0.48–1.25)	0.353
25(OH)VD, n (%)						
<30.00 ng/mL	1,527 (75.0)	692 (74.0)	268 (75.1)	396 (71.6)	17 (89.1)	<0.001
≥30.00 ng/mL	510 (25.0)	243 (26.0)	89 (24.9)	157 (28.4)	21 (10.9)	
TgAb, n (%)						
<15.00 IU/mL (undetectable)	1,931 (94.8)	874 (93.5)	340 (95.2)	528 (95.5)	189 (98.4)^a^	0.028
15.00–60.00 IU/mL	106 (5.2)	61 (6.5)	17 (4.8)	25 (4.5)	3 (1.6)	0.028
TPOAb, n (%)						
<28.00 IU/mL (undetectable)	1,187 (58.3)	539 (57.6)	206 (57.7)	331 (59.9)	111 (57.8)	0.853
28.00–60.00 IU/mL	850 (41.7)	396 (42.4)	151 (42.3)	222 (40.1)	81 (42.2)	0.853
Season of blood draw						0.611
Summer	1,144 (56.2)	524 (56.0)	190 (53.2)	313 (56.6)	117 (60.9)	
Autumn	539 (26.5)	255 (27.3)	97 (27.2)	145 (26.2)	42 (21.9)	
Winter	354 (17.4)	156 (16.7)	70 (19.6)	95 (17.2)	33 (17.2)	

**Notes.**

A one-way analysis of variance (ANOVA) was used to compare free triiodothyronine (FT3) and free thyroxine (FT4) levels across C-TIRADS groups, with pairwise comparisons conducted using the Bonferroni correction method. The non-parametric Kruskal–Wallis *H* test was applied to analyze differences in age, body mass index (BMI), thyroid-stimulating hormone (TSH), 25-hydroxy vitamin D [25(OH)VD], 25(OH)VD3, and 25(OH)VD2 levels across C-TIRADS groups. Multiple comparisons were performed using the Bonferroni correction method. Pearson’s chi-squared test was used to analyze differences in thyroglobulin antibody (TgAb), thyroperoxidase antibody (TPOAb) levels, and season of blood draw. Significant differences were identified when comparing with the reference group (*e.g.*, a: compared with C-TIRADS1, *p* < 0.05; b: compared with C-TIRADS2, *p* < 0.05; c: compared with C-TIRADS3, *p* < 0.05).

Abbreviations C-TIRADS Chinese-Thyroid Imaging Reporting and Data System TNs thyroid nodules

### Distribution of TNs

To further investigate associations between VitD levels and TN risk, serum concentrations of 25(OH)VD, 25(OH)VD3, and 25(OH)VD2 were stratified into low (L), medium (M), and high (H) tertiles. No significant differences in overall TN prevalence were observed across 25(OH)VD groups (VD-L: 53.8% *vs.* VD-M: 55.4% *vs.* VD-H: 52.4%; *p* = 0.546; Fig. S1A). Similarly, neither 25(OH)VD3 (VD3-L: 54.2% *vs.* VD3-M: 56.0% *vs.* VD3-H: 52.1%; *p* = 0.366; Fig. S1B) nor 25(OH)VD2 (VD2-L: 53.9% *vs.* VD2-M: 57.4% *vs.* VD2-H: 51.0%; *p* = 0.055; Fig. S1C) groups showed significant differences in overall TN prevalence.

**Figure 2:**
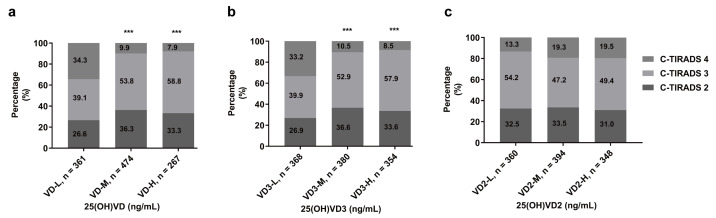
Distribution of C-TIRADS classifications for TNs among petroleum workers with TNs (*n* = 1102). The Pearson chi-square test was used to evaluate differences in TN distribution across categories of serum (A) 25(OH)VD, (B) 25(OH)VD3, or (C) 25(OH)VD2 levels. Serum 25(OH)VD levels were categorized based on international and domestic guidelines for Vit D nutritional status, while 25(OH)VD3 and 25(OH)VD2 levels were divided into tertiles. ***p < 0.001 compared with the VD-L group or VD3-L group. Abbreviations are as defined in Table 1.

When subclassifying the 1,102 TN patients by C-TIRADS, significant distributional variations emerged among C-TIRADS categories (2, 3, and 4) for 25(OH)VD (*p* < 0.001; Fig. 2A) and 25(OH)VD3 groups (*p* < 0.001; Fig. 2B). In contrast, 25(OH)VD2 groups exhibited no significant distributional differences (*p* = 0.118; Fig. 2C). Notably, the low 25(OH)VD group demonstrated a significantly higher proportion of C-TIRADS 4 TNs (34.3%) compared to medium (9.9%; *P*_adj_ < 0.001) and high groups (7.9%; *P*_adj_ < 0.001), with no significant difference between medium and high groups (*P*_adj_ = 0.427; Table 2). Parallel results were observed for 25(OH)VD3: the low group had a higher proportion of C-TIRADS 4 TNs (33.2%) than medium (10.5%; P_adj_ < 0.001) and high groups (8.5%; *P*_adj_ < 0.001), while medium *versus* high groups showed no significant difference (*P*_adj_ = 0.380; Table 2).

**Table 2:** TN distribution patterns in petroleum workers according to serum VitD Levels.

TNs	25(OH)VD (ng/mL)			25(OH)VD3 (ng/mL)		
	VD-L (<20.00)	VD-M (20.00–30.0)	VD-H (≥ 30.00)	VD3-L (<19.07)	VD3-M (19.07–26.70)	VD3-H (≥26.70)
C-TIRADS 2 n (%)	96^a^ (26.6)	172^b^ (36.3)	8996^a,b^ (33.3)	99^a^ (26.9)	139^b^ (36.6)	119^a,b^ (33.6)
C-TIRADS 3 n (%)	141^a^ (39.1)	255^b^ (53.8)	157^b^ (58.8)	147^a^ (39.9)	20^b^ (52.9)	205^b^ (57.9)
C- TIRADS 4 n (%)	124^a^ (34.3)	47^b^ (9.9)	21^b^ (7.9)	122^a^ (33.2)	40^b^ (10.5)	30^b^ (8.5)
Total n (%)	361 (100.0)	474 (100.0)	267 (100.0)	368 (100.0)	380 (100.0)	354 (100.0)

**Notes.**

Among the petroleum workers with TNs (*n* = 1102), differences in the distribution of TN categories across the L, M, and H groups of 25(OH)VD and 25(OH)VD3 were analyzed using the Bonferroni correction method. Superscripts sharing the same letter indicate that the percentages of TN in the same category were not significantly different between the L, M, and H groups of 25(OH)VD or 25(OH)VD3. The stratification of VitD levels was performed as described in Fig. 2. Abbreviations are defined in Table 1.

### Prevalence of C-TIRADS 4 TNs

Analysis of 2,037 petroleum workers revealed significantly higher prevalence of C-TIRADS 4 TNs in the 25(OH)VD-L group (18.5%) compared to the VD-M group (5.5%; *p* < 0.001) and VD-H group (4.1%; *p* < 0.001). No significant difference was observed between the VD-M and VD-H groups (*p* = 0.304; Fig. S2A). Analogous patterns occurred in the 25(OH)VD3 groups: VD3-L demonstrated higher prevalence (18.0%) than VD3-M (5.9%; *p* < 0.001) and VD3-H (4.4%; *p* < 0.001; Fig. S2B).

To evaluate the likelihood of developing C-TIRADS 4 TNs among petroleum workers with varying serum 25(OH)VD levels, binary logistic regression analyses were performed. Models were adjusted for established confounders, including age (Haymart, 2009) , BMI (Kim, Gosnell & Roman, 2020), thyroid function (Kitahara et al., 2024), and thyroid autoantibodies (Kitahara et al., 2024), given their documented associations with thyroid cancer. Additionally, to account for potential confounding by seasonal fluctuations in VitD synthesis (Holick et al., 2011), the season of blood draw was incorporated as a covariate in multivariable logistic regression models. Results demonstrated that the VD-L group had a 5.28-fold increased risk of developing C-TIRADS 4 TNs *versus* the VD-H group (adjusted OR = 5.28; 95% CI [3.27–8.52]; *p* < 0.001). Conversely, the VD-M group showed no significant risk elevation relative to the VD-H group (adjusted OR = 1.35; 95% CI [0.80–2.30]; *p* = 0.261) (Table 3). Parallel trends were observed for 25(OH)VD3 levels: the VD3-L group had an adjusted OR of 4.74 (95% CI [3.13–7.18]; *P* < 0.001) and the VD3-M group 1.35 (95% CI [0.83–2.20]; *P* = 0.221) relative to VD3-H (Table 3). Due to non-significant risk differences between VD-M and VD-H groups, 25(OH)VD levels were stratified at 20.00 ng/mL. After full adjustment, individuals with serum 25(OH)VD < 20.00 ng/mL had a 4.07-fold higher risk of developing C-TIRADS 4 TNs *versus* those with levels ≥ 20 ng/mL (adjusted OR = 4.07; 95% CI [2.96–5.61]; *p* < 0.001; data not shown).

**Table 3:** Binary logistic regression assessing the risk of C-TIRASD 4 TN prevalence in 2,037 petroleum workers stratified by serum 25(OH)VD or 25(OH)VD3 levels.

VitD status	Unadjusted		Model 1		Model 2	
	OR (95% CI)	*p*-value	OR (95% CI)	*p*-value	OR (95% CI)	*p*-value
25(OH)VD, ng/mL						
VD-L (<20.00 )	5.28 (3.27–8.52)	<0.001	5.25 (3.25–8.51)	<0.001	5.07 (3.11–8.26)	<0.001
VD-M (20.00–30.00)	1.35 (0.80-2.30)	0.261	1.40 (0.83–2.38)	0.211	1.36 (0.80–2.33)	0.256
VD-H (≥ 30.00)^#^	1.00		1.00		1.00	
25(OH)VD3, ng/mL						
VD3-L (<19.07)	4.74 (3.13–7.18)	<0.001	4.72 (3.01–7.18)	<0.001	4.56 (2.98–6.98)	<0.001
VD3-M (19.07–26.70)	1.35 (0.83–2.20)	0.221	1.42 (0.87–2.32)	0.160	1.38 (0.84–2.26)	0.200
VD3-H(≥ 26.70)^#^	1.00		1.00		1.00	

**Notes.**

Model 1 was adjusted for age and BMI.

Model 2 was adjusted for age, BMI, FT3, FT4, TSH, TPOAb, TgAb and season of blood draw.

# The reference group.

The stratification of serum VitD levels was performed as described in Fig. 2.

## Discussion

This study investigated the association between VitD nutritional status and the prevalence of TNs, particularly those with malignant tendency (C-TIRADS 4), in a cohort of 2037 petroleum workers using health check-up data. Analysis revealed that patients with C-TIRADS 4 TNs exhibited significantly lower serum 25(OH)VD and 25(OH)VD3 levels compared to other classifications, while 25(OH)VD2 remained unchanged—aligning with prior research on total VitD (Fan et al., 2022), while newly highlighting the differential involvement of VitD3 and VitD2. Stratification by serum concentrations (low/medium/high) demonstrated no significant differences in overall TN prevalence across 25(OH)VD, 25(OH)VD3, or 25(OH)VD2 groups. Crucially, within the TN subgroup (*n* = 1102), petroleum workers with low 25(OH)VD or 25(OH)VD3 levels showed a significantly higher proportion of C-TIRADS 4 TNs, an effect absent for 25(OH)VD2. Binary logistic regression, after adjusting for confounders, revealed that low 25(OH)VD or 25(OH)VD3 levels were independently associated with C-TIRADS 4 TNs. Stratified analysis further identified that 25(OH)VD <20.00 ng/mL was independently associated with C-TIRADS 4 TNs. These findings suggest that reduced VitD3 (but not VitD2) levels may not be associated with overall TN prevalence in petroleum workers but are correlated with an increased likelihood of TNs with malignant tendency. Future investigations into the VitD-TN relationship should prioritize 25(OH)VD3 to validate these preliminary observational findings.

Emerging evidence suggests VitD and its analogs may exert protective effects against thyroid tumorigenesis. Proposed mechanisms include inducing cell cycle arrest, inhibiting proliferation, and promoting apoptosis. Specifically, VitD has been shown to induce p27 dephosphorylation and accumulation *via* both PTEN/AKT-dependent and -independent pathways, leading to G1 phase arrest in thyroid cancer stem cells (CSCs) (Liu et al., 2002; Dackiw et al., 2004). Furthermore, VitD can suppress the Ras-MEK-ERK pathway, upregulate pro-apoptotic Caspase-3 expression (both protein and mRNA), and downregulate the proliferation marker Ki67 (Li, Lv & Li, 2020). Crucially, these VitD effects are predominantly mediated by its receptor, the VDR, which exerts growth-inhibitory effects in differentiated thyroid cancer cells by modulating the E-cadherin/β-catenin complex. This suggests that targeting the VDR pathway represents a promising therapeutic strategy against thyroid cancer progression (Bains et al., 2021; Ling et al., 2022). However, the precise mechanisms underlying these effects remain incompletely understood, and conflicting evidence exists (Palanca, Ampudia-Blasco & Real, 2022; Lawler & Warren Andersen, 2023). For instance, Kuang et al. (2022) found no reliable evidence linking serum VitD levels to the risk or prognosis of PTC when comparing PTC patients to those with benign TNs.

Several factors may explain these discrepancies. Firstly, prior studies typically measured only total serum VitD without distinguishing between the individual contributions of VitD3 and VitD2. Secondly, inconsistencies in study population selection across existing research contribute to divergent conclusions regarding the VitD-TN relationship. Thirdly, the use of varied methodologies and units for quantifying serum VitD complicates comparisons. Finally, immunological assays commonly employed in earlier studies exhibit cross-reactivity between VitD3 and VitD2 metabolites and are less accurate than LC-MS/MS methods (Roth et al., 2008; Lai et al., 2012; Haznadar et al., 2018).

Research directly comparing the mechanisms of action between VitD2 and VitD3 is limited. One study demonstrated that VitD3, unlike VitD2 , significantly enhanced the immune response of Atlantic salmon primary macrophages to bacterial infections (Soto-Dávila et al., 2019). This effect was mediated by VitD3-dependent upregulation of leukocyte-derived chemotaxin 2 (Lect-2), promoting neutrophil recruitment and augmenting antimicrobial immunity (Soto-Dávila et al., 2019). This indicates a more potent immunomodulatory role for VitD3. Given evidence that loss of MHC-I (Major Histocompatibility Complex class I) expression in PTC facilitates tumor immune evasion—a deficiency reversible by interferon-alpha (IFN-α), leading to enhanced immune cytotoxicity and tumor growth inhibition (Angell et al., 2014; Motylewska et al., 2014)—and the finding that VitD3 (but not VitD2) enhances IFN-α response gene expression (Durrant et al., 2022), we hypothesize that VitD3 may promote T cell-mediated anti-tumor immunity. This could occur *via* upregulation of MHC-I expression on thyroid tumor cells through enhanced IFN-*α*-regulated gene activity. This proposed mechanism may partially explain our observation that deficiency in VitD3, but not VitD2, is statistically associated with C-TIRADS 4 TNs in this retrospective cohort. However, this proposed immunomodulatory mechanism remains speculative, as it has not been directly validated by our observational data; definitive validation would require dedicated *in vitro* studies investigating the effects of VitD3 on immune cells involved in thyroid cancer progression, alongside well-designed *in vivo* animal models.

Interpreting the observed associations within this specific cohort of petroleum workers requires careful consideration of potential confounding by occupational exposures. Petroleum workers are routinely occupationally exposed to complex mixtures present in crude oil, its derivatives, and processing environments, including volatile organic compounds (VOCs) such as benzene, toluene, ethylbenzene, and xylene (BTEX), polycyclic aromatic hydrocarbons (PAHs), and heavy metals (Fowles et al., 2016; Zhou et al., 2023). These substances exhibit well-characterized mutagenic, carcinogenic, and endocrine-disrupting properties (Fowles et al., 2016; Lang & Beier, 2018; Abraham & Li, 2022), prompting a critical question: Could these occupational exposures—either independently or in combination with VitD deficiency—contribute to thyroid cancer risk, thereby confounding the observed association between VitD deficiency and thyroid cancer?

Occupational exposures may directly drive thyroid cancer risk, potentially masking the true role of VitD deficiency. Experimental and epidemiological studies support direct thyroid toxicity of these exposures: for instance, toluene exposure increases tumor incidence in specific organs of experimental animals (Dees, Askari & Henley, 1996; Maltoni et al., 1997), while occupational exposure to mixed solvents (including toluene) correlates with elevated thyroid cancer risk (Kim et al., 2021). Additionally, benzene and formaldehyde exposure has been linked to increased thyroid cancer incidence in female textile workers (Wong et al., 2006), with multiple lines of evidence supporting an association between VOCs exposure and thyroid cancer (Guo et al., 2015; Protano et al., 2021; Rosol & Witorsch, 2021).

Occupational exposures may also confound the causal association between VitD deficiency and thyroid cancer by disrupting VitD metabolism, potentially rendering “VitD deficiency” a concomitant consequence of exposure rather than an independent risk factor. First, chronic chemical exposure (*e.g.*, VOCs, heavy metals) can disrupt VitD synthesis and metabolism through mechanisms such as oxidative stress, thereby reducing VitD levels (Li et al., 2023; Hu et al., 2024). In such cases, the observed “VitD deficiency” may reflect a biological effect of occupational exposure rather than a causal factor for thyroid cancer. Second, VOC exposure is associated with comorbidities including obesity, metabolic syndrome, and kidney disease—conditions established as risk factors for VitD deficiency (Abraham & Li, 2022; Lei et al., 2023; Dong et al., 2024; Wu et al., 2024). Third, heavy metals, per- and polyfluoroalkyl substances (PFAS), and other pollutants downregulate α-Klotho expression; as a critical regulatory protein in VitD metabolism and calcium homeostasis, reduced α-Klotho expression may thereby indirectly contribute to VitD deficiency (Abraham & Li, 2022; Jain & Ducatman, 2022; Kim et al., 2022; Yao et al., 2022; Li et al., 2023).

Therefore, it is imperative to recognize these occupational exposures (*e.g.*, BTEX, PAHs, heavy metals) as significant potential confounding factors. They may independently affect both VitD metabolic pathways and thyroid pathophysiology, thereby potentially confounding the observed association between VitD3 deficiency and C-TIRADS 4 nodules in this specific working population. Future studies should aim to enroll more diverse populations and incorporate quantitative assessments of specific occupational and environmental chemical exposures to further validate the findings presented here and disentangle the complex interplay among VitD status, chemical exposures, and thyroid cancer risk.

This study focused exclusively euthyroid male petroleum workers aged 30–60 years. This age range was selected primarily to align with the study’s objective of investigating the association between VitD status and TNs in a core occupational cohort with significant occupational exposures. Workers aged 30–60 years are typically the main workforce in the petroleum industry and are at a life stage with the highest prevalence of thyroid cancer (Belfiore et al., 1992). Excluding individuals younger than 30 years was based on the lower prevalence of TNs in this group (Belfiore et al., 1992) and typically shorter durations of occupational exposure, which might limit the assessment of exposure-outcome relationships. Exclusion of those older than 60 years aimed to minimize confounding from age-related comorbidities (*e.g.*, cardiovascular disease, diabetes, other endocrine disorders), altered medication use, potential retirement or job role changes reducing current exposure levels, and the profound physiological changes associated with aging that independently affect both VitD metabolism and thyroid cancer development (Haitchi, Moliterno & Widhalm, 2023).

While this selection enhances internal validity by providing a stable, exposed cohort with fewer competing risks, it strictly limits generalizability: our findings are only applicable to this specific subgroup, and caution is warranted when extending results to younger/older petroleum workers, female workers, or individuals in other occupations, highlighting the need for future studies encompassing broader age ranges (including longitudinal assessments from career inception to retirement) and female workers to explore age/gender modifiers and validate generalizability. Additionally, the retrospective design introduces inherent limitations, most notably selection bias: data derived from voluntary health check-up records may overrepresent health-conscious individuals or those with preexisting concerns, potentially skewing observed TN prevalence and VitD deficiency rates. Further, petroleum workers in specialized roles (*e.g.*, outdoor *vs.* indoor) may differ in sunlight exposure (a key determinant of VitD synthesis) or occupational toxin exposure, which could independently impact thyroid health but were not fully accounted for. Importantly, we lacked histological confirmation of TN type, relying instead on C-TIRADS, which is less definitive than biopsy. For instance, C-TIRADS 4 nodules may include benign nodules misclassified as high-risk or malignant nodules misclassified as low-risk, weakening potential associations between VitD and cancer risk. Even with dual radiologist review, ultrasound assessments are subject to interobserver variability (due to equipment, expertise, or criterion application), further introducing misclassification bias. These factors should be considered when interpreting our findings.

## Conclusion

In euthyroid male petroleum workers without TAI, VitD3 deficiency [25(OH)D3 <19.07 ng/mL], but not VitD2, was independently associated with an increased risk of TNs exhibiting malignant ultrasound features. However, given the retrospective cohort design of this study, further validation through prospective cohort studies with larger sample sizes and functional experiments is warranted to establish the potential independent causal relationship.

## Supplemental Information

10.7717/peerj.20893/supp-1 Supplemental Information 1 Raw data

10.7717/peerj.20893/supp-2 Supplemental Information 2 Prevalence of TNs among all 2307 petroleum workers, stratified by serum Vit D levels Differences in TN prevalence among the low (L), medium (M), and high (H) groups of 25(OH)VD, 25(OH)VD3, and 25(OH)VD2 were analyzed using chi-square tests. Abbreviations are as described in tabreftab1.

10.7717/peerj.20893/supp-3 Supplemental Information 3 Prevalence of C-TIRADS 4 TNs among all 2037 petroleum workers, stratified by serum Vit D levels The Pearson chi-square test was used to assess differences in the prevalence of C-TIRADS 4 TNs across the low (L), medium (M), and high (H) groups of 25(OH)VD or 25(OH)VD3. The adjusted p-value (Padj) was calculated by multiplying the original p-value by 3. A Cochran–Mantel–Haenszel test was applied to examine trends in the prevalence of C-TIRADS 4 TNs according to serum 25(OH)VD or 25(OH)VD3 levels. Abbreviations are as defined in Table 1.

## Additional Information and Declarations

### Data Availability

The following information was supplied regarding data availability: The raw measurements are available in the Supplemental File.
