# Supplementary figures and images for: Association of VitD 3 deficiency with thyroid nodules suspected of malignancy in petroleum workers: a retrospective cohort study
Source: PeerJ. 2026 Feb 27;14:e20893. doi: 10.7717/peerj.20893 (PMC12951886; doi:10.7717/peerj.20893)

**a**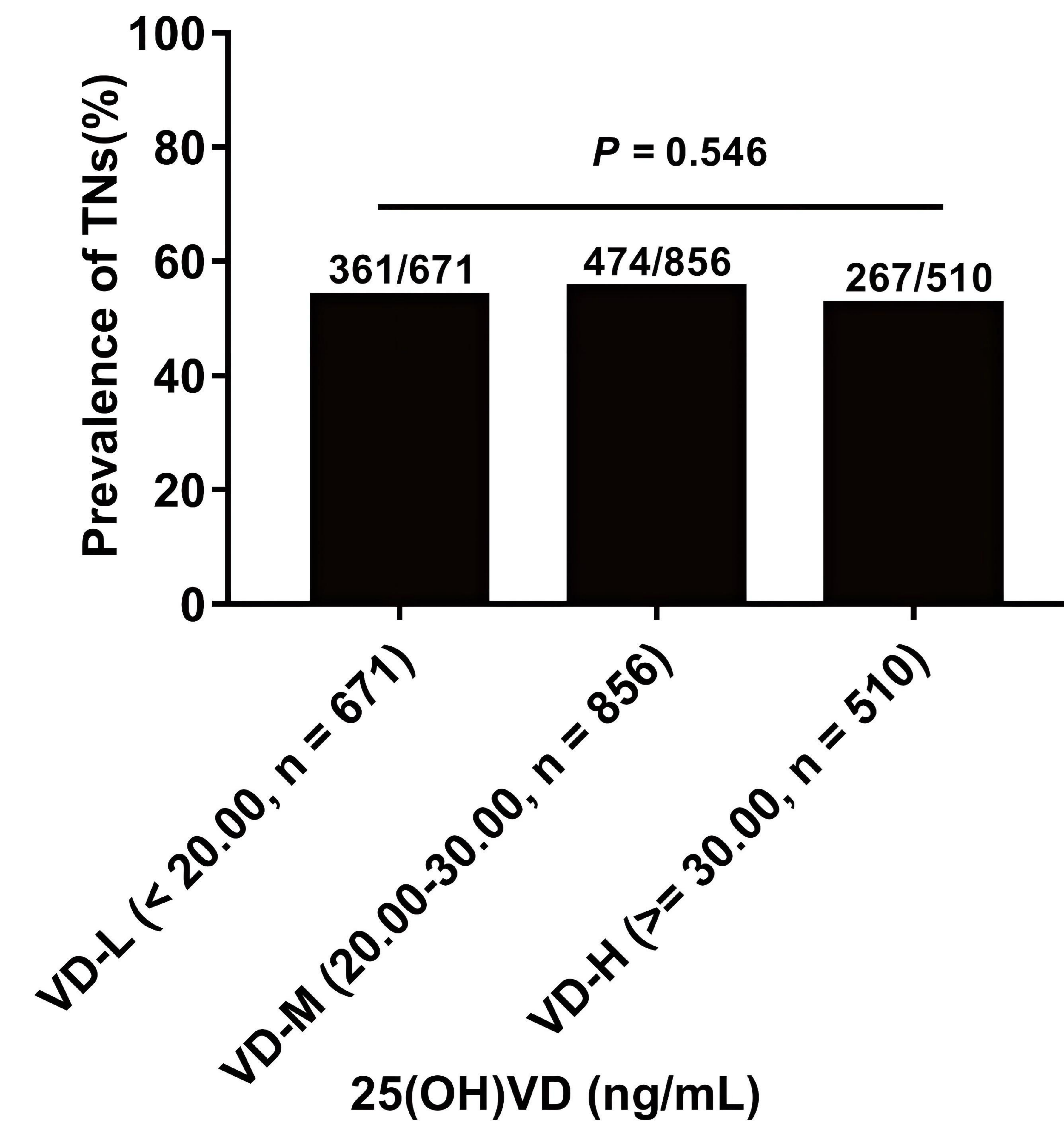**b**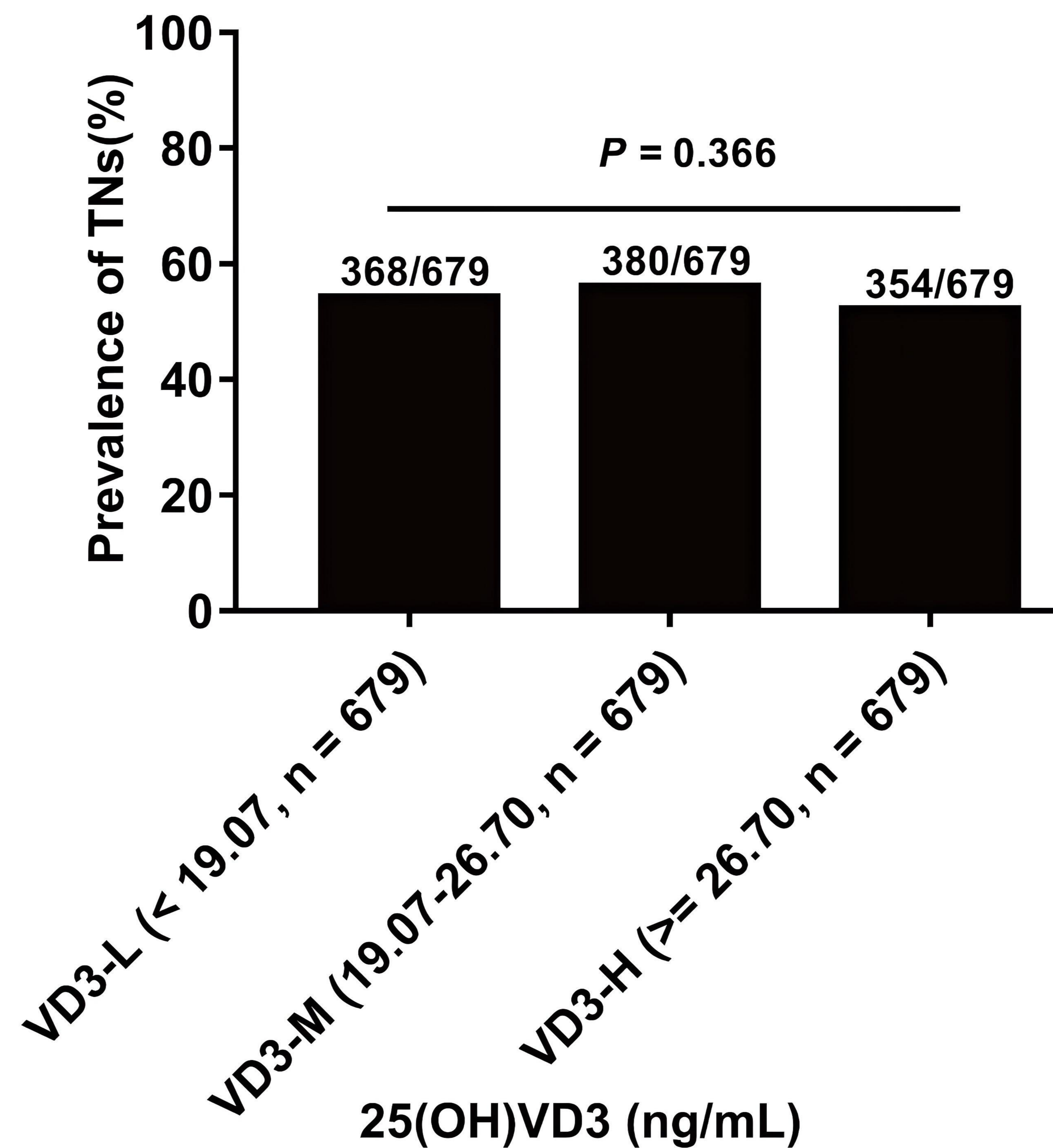**c**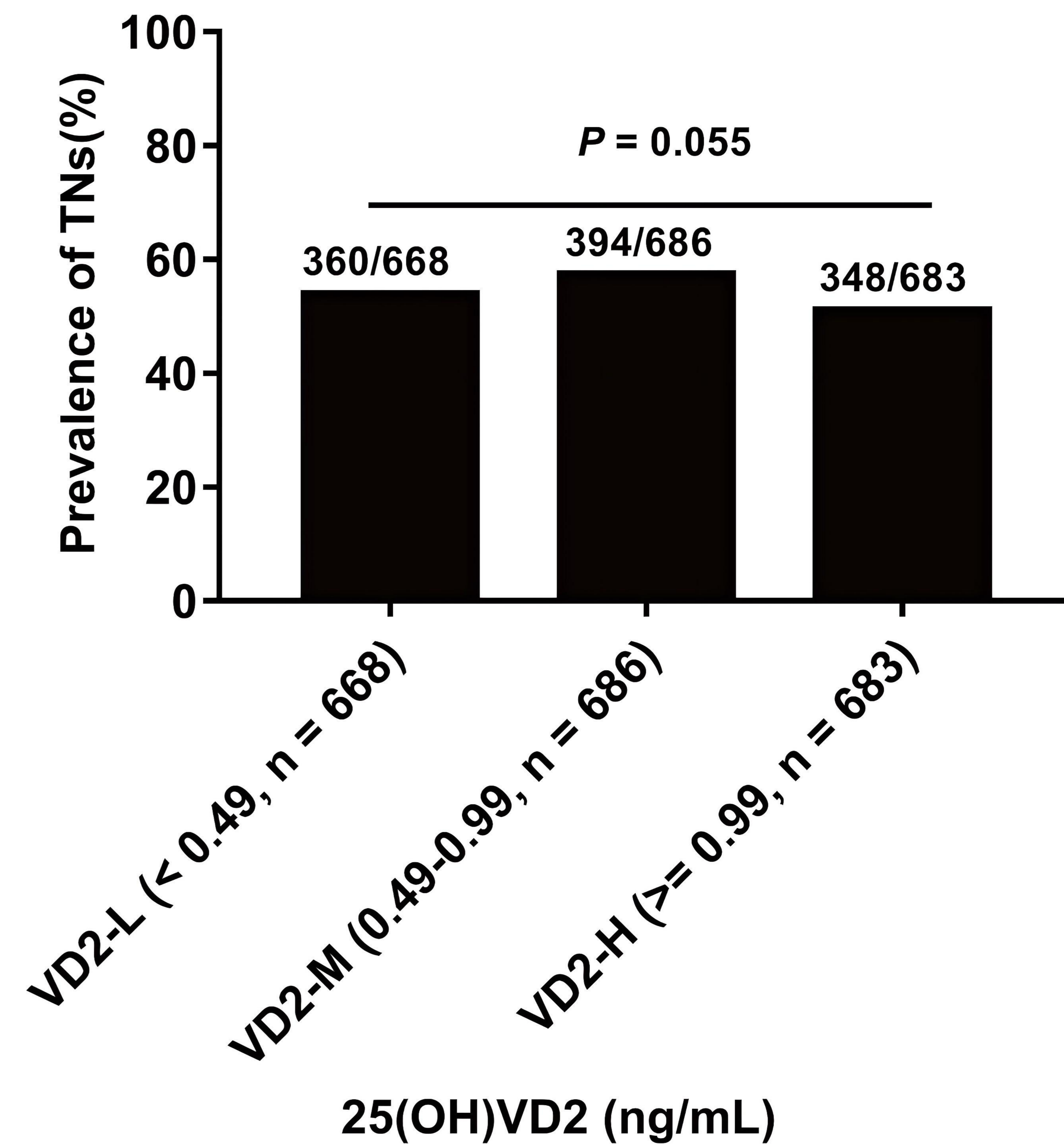

Supplement: Supplemental Information 2 — Differences in TN prevalence among the low (L), medium (M), and high (H) groups of 25(OH)VD, 25(OH)VD3, and 25(OH)VD2 were analyzed using chi-square tests. Abbreviations are as described in tabreftab1. [file peerj-14-20893-s002.pdf]

**a**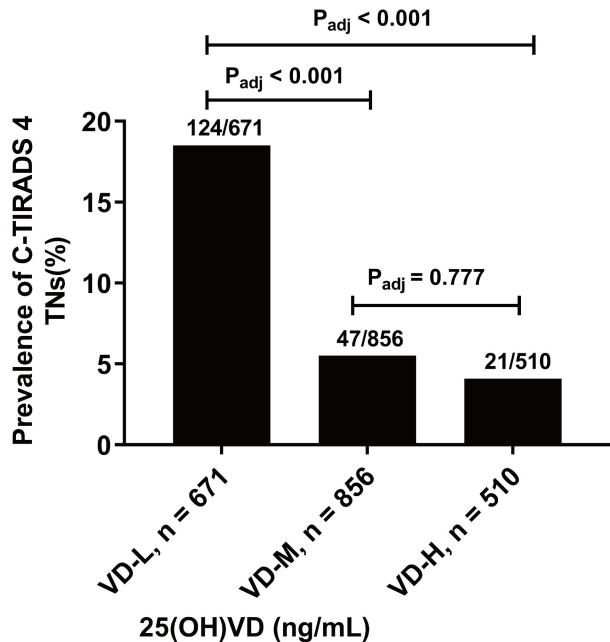**b**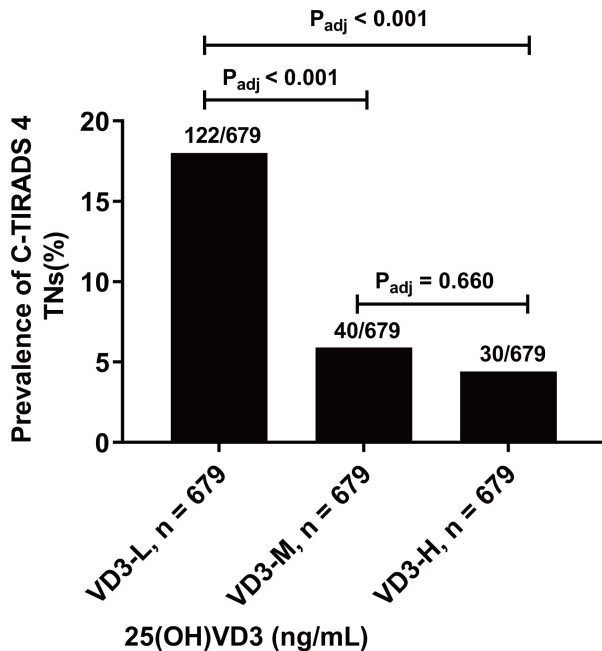

Supplement: Supplemental Information 3 — The Pearson chi-square test was used to assess differences in the prevalence of C-TIRADS 4 TNs across the low (L), medium (M), and high (H) groups of 25(OH)VD or 25(OH)VD3. The adjusted p-value (Padj) was calculated by multiplying the original p-value by 3. A Cochran–Mantel–Haenszel test was applied to examine trends in the prevalence of C-TIRADS 4 TNs according to serum 25(OH)VD or 25(OH)VD3 levels. Abbreviations are as defined in Table 1. [file peerj-14-20893-s003.pdf]
